# Expression pattern of the urokinase-plasminogen activator system in rat DS-sarcoma: Role of oxygenation status and tumour size

**DOI:** 10.1038/sj.bjc.6600237

**Published:** 2002-04-22

**Authors:** M Weinmann, O Thews, T Schroeder, P Vaupel

**Affiliations:** Institute of Physiology & Pathophysiology, University of Mainz, 55099 Mainz, Germany

**Keywords:** urokinase plasminogen activator, plasminogen activator inhibitor type-1, urokinase plasminogen activator receptor, hypoxia, DS-sarcoma, malignant progression

## Abstract

The urokinase plasminogen activator system plays a central role in malignant tumour progression. Both tumour hypoxia and enhancement of urokinase plasminogen activator, urokinase plasminogen activator-receptor and plasminogen activator inhibitor type 1 have been identified as adverse prognostic factors. Upregulation of urokinase plasminogen activator or plasminogen activator inhibitor type 1 could present means by which hypoxia influences malignant progression. Therefore, the impact of hypoxia on the expression pattern of the urokinase plasminogen activator system in rat DS-sarcoma *in vivo* and *in vitro* was examined. In the *in vivo* setting, tumour cells were implanted subcutaneously into rats, which were housed under either hypoxia, atmospheric air or hyperoxia. For *in vitro* studies, DS-sarcoma cells were incubated for 24 h under hypoxia. Urokinase plasminogen activator and urokinase plasminogen activator-receptor expression were analysed by flow cytometry. Urokinase plasminogen activator activity was measured using zymography. Plasminogen activator inhibitor type 1 protein levels *in vitro* and *in vivo* were examined with ELISA. PAI-1 mRNA levels were determined by RT–PCR. DS-sarcoma cells express urokinase plasminogen activator, urokinase plasminogen activator-receptor, and plasminogen activator inhibitor type 1 *in vitro* and *in vivo*. The urokinase plasminogen activator activity is enhanced in DS-sarcomas compared to normal tissues and rises with increasing tumour volume. The oxygenation level has no impact on the urokinase plasminogen activator activity in cultured DS-sarcoma cells or in solid tumours, although *in vitro* an increase in plasminogen activator inhibitor type 1 protein and mRNA expression after hypoxic challenge is detectable. The latter plasminogen activator inhibitor type 1 changes were not detectable *in vivo*. Hypoxia has been demonstrated to contribute to the upregulation of some components of the system *in vitro*, although this effect was not reproducible *in vivo*. This may indicate that the serum level of plasminogen activator inhibitor type 1 is not a reliable surrogate marker of tumour hypoxia.

*British Journal of Cancer* (2002) **86**, 1355–1361. DOI: 10.1038/sj/bjc/6600237 www.bjcancer.com

© 2002 Cancer Research UK

## 

Tumour invasion, malignant progression and distant metastasis depend on complex multistep processes. One prerequisite is the ability of tumour cells to initiate extracellular proteolysis which is required for the crossing of tissue barriers, migration, invasion, tissue remodelling and angiogenesis. There is abundant experimental evidence that serine proteases of the urokinase-plasminogen activator system play a major role in malignant progression and tumour metastasis (Andreasen and Petersen, 2000).

The urokinase-plasminogen activator (uPA) is secreted in a zymogen form as inactive single-chain proenzyme (pro-uPA) by tumour and/or by stromal cells and is converted by limited proteolytic cleavage into the active two-chain enzyme. It binds to the urokinase plasminogen activator receptor (uPAR), which is located on the surface of tumour cells, and also on cells of the tumour stroma. The binding to the receptor leads to a massive enhancement of proteolytic activity, providing an efficient and locally focussed proteolysis. The main target of uPA is plasminogen, which is converted into plasmin. Urokinase-plasminogen activator is also involved in the activation of hepatocyte growth factor/scatter factor (HGF/SF). Plasmin is able to cleave a variety of proteins of the extracellular matrix (ECM) such as collagen type IV, laminin, fibronectin and vitronectin and is also involved in the conversion of some matrix-metalloproteinases from the zymogen pro-form into active enzymes and in the activation of latent TGFβ or in the release of bFGF from ECM-binding sites.

The proteolytic activity of the uPA/uPAR complex is regulated by so-called serpins (plasminogen activator inhibitor type I (PAI-1) and type II (PAI-2)). The binding of PAI induces the internalisation of the uPA/uPAR complex, intracellular degradation of uPA and the recycling of the uPAR to the cell membrane (for a review see Andreasen *et al*, 1997).

An upregulation of uPA and/or uPAR has been described for many human tumours. High levels of uPA and uPAR in tumour tissue (and also in plasma) are associated with poor prognosis of patients with cancers of the breast, head and neck, uterine cervix, bladder, lung, ovary or with soft tissue sarcoma (for a review see Reuning *et al*, 1998). Paradoxically, many studies also identified enhanced levels of PAI-1, but not PAI-2, as an adverse prognostic factor (Reuning *et al*, 1998). The role of PAI-1 in tumour progression is not yet fully understood. Additional functions beyond the involvement in proteolysis may explain the tumour-promoting role of PAI-1 (Bajou *et al*, 1998).

So far, the events leading to an enhanced expression of uPA, uPAR or PAI-1 in solid tumours have not been fully elucidated. Cytokines and oncogenes are able to induce components of the uPA-system (Reuning *et al*, 1998), but the trigger mechanisms for upregulation could also be derived from the specific tumour micro-environment.

Most tumours are characterised by low extracellular pH, glucose depletion, high lactate levels and regions with low oxygen tensions (Vaupel *et al*, 1989, 1992, 1998). Low oxygen tensions in human tumours have been associated with poor outcome (e.g. Höckel *et al*, 1993, 1996; Nordsmark and Overgaard, 2000; Nordsmark *et al*, 2001), enhanced local or locoregional spread (Höckel *et al*, 1999) and enhanced metastatic potential (Brizel *et al*, 1996; Sundfor and Rofstad, 1998; Rofstad, 2000).

Tumour hypoxia is a key parameter, able to modulate the expression of a variety of genes which are involved in tumour angiogenesis, malignant progression and distant metastasis (Dachs and Tozer, 2000; Koong *et al*, 2000; Höckel and Vaupel, 2001). Some components of the uPA-system have also been shown to be induced by hypoxia in a number of cell lines *in vitro* (Graham *et al*, 1998, 1999; Kietzmann *et al*, 1999; Maity and Solomon, 2000).

The aim of this study is the characterisation of the expression of uPA, uPAR and PAI-1 in rat DS-sarcoma *in vitro* and *in vivo* (i.e., from tumour cell culture to the solid tumour), the identification of the main cellular source in solid DS-tumours and the examination of the dependency of uPA, uPAR and PAI-1 expression on micro-environmental factors such as oxygenation status.

## MATERIALS AND METHODS

### Animals and tumours

0.4 ml of haemorrhagic ascites of syngenic DS-sarcoma cells (10^4^ cells μl^−1^) were injected s.c. into the hind foot dorsum of Sprague Dawley rats (6–8 weeks of age, body weight 180–200 g, Charles River, Sulzfeld, Germany). Tumours were measured daily using precision callipers, and were excised once a volume of between 1 and 2 ml was reached (for experiments evaluating the impact of volume, tumours between 0.5 and 3.5 ml were used). Immediately after excision single cell suspensions were prepared for flow cytometry, or alternately tumours were frozen in liquid nitrogen and stored at –80°C. For experiments examining the role of the oxygenation status, animals were housed during the whole period of tumour growth in a hypoxic atmosphere (8% O_2_/92% N_2_), normal room air or under hyperoxic conditions (100% O_2_) for 7–12 days. All animal experiments performed had been approved by the regional ethics committee according to German federal law. The ethical guidelines that were followed meet the standards required by the ‘United Kingdom Committee on Cancer Research (UKCCCR) guidelines for the welfare of animals in experimental neoplasia’ (Workman *et al*, 1998).

### Hypoxic cell culture

To establish hypoxic conditions *in vitro*, a gas-proof stainless steel chamber inside an incubator was flushed with a prewarmed and water saturated gas mixture of ≈95% N_2_/≈5% CO_2_ and <0.1% O_2_. After an initial peak, the gas flow was adjusted to 0.5 l h^−1^ and the time course of oxygen washout was measured. DS-sarcoma cells were isolated from solid tumours and grown *in vitro*. For hypoxic challenge of DS-sarcoma cells, three petri dishes (2×10^5^ cells ml^−1^ medium, Petri Perm™, Heraeus, Hanau, Germany) were placed into the hypoxic chamber for 24 h. The hypoxic period commenced (t_0_=0 h) when the gas flow was started. Under these conditions, after a wash out period of 2 h, the O_2_ concentration in the chamber was below 1%. Control cells were incubated under a normal atmospheric oxygen concentration (H_2_O saturated room air, 5% CO_2_).

### Indirect flow cytometry of uPA and uPAR

Single cell suspensions of solid tumours were prepared and contaminating red blood cells removed using an RBC lysis buffer. The suspension was adjusted to give a concentration of 0.5–1×10^6^ cells ml^−1^ in phosphate buffered saline supplemented with 1% glucose, washed twice and stained with either uPA (sc-6831 Santa Cruz Biotech., Santa Cruz, CA, USA), uPAR polyclonal IgG antibody (sc-9795 Santa Cruz Biotech., Santa Cruz, CA, USA) or normal goat IgG antibody (sc-2028 Santa Cruz Biotech., Santa Cruz, CA, USA) as negative controls. Both antibodies were detected with a FITC-labelled donkey anti-goat IgG antibody (sc-2024 Santa Cruz Biotech., Santa Cruz, CA, USA) and measured using flow cytometry (Coulter EPICS Profile, Beckman Coulter, Palo Alto, CA, USA).

### One-phase uPA zymography

Whole cell protein extracts were prepared from 20 μm cryosections of solid tumours or from washed cell suspensions by incubating the homogenised cell material for 45 min at 4°C in a lysis buffer (0.01 M HEPES, 0.25 M Sucrose, 0.5% v v^−1^, Triton X-100, pH 7.2). Debris was removed by centrifugation (15 000×*g*) for 15 min at 4°C and supernatants were stored at −80°C. Protein content was determined by a DC protein assay (BioRad, Hercules, CA, USA) and adjusted to 1 mg ml^−1^ for zymography. Seven μl of cell lysates or cell culture supernatants were run on a 10% SDS–PAGE containing 0.3% casein and 0.025 U ml^−1^ Lys-plasminogen under non-reducing conditions together with prestained molecular weight markers (BioRad, Hercules, CA, USA). Gels were washed after electrophoresis in a washing buffer (Triton X-100 2.5%, 0.1 M TRIS–HCl, pH 8.1) for 2×15 min and incubated overnight in 10 mM CaCl_2_, 50 mM TRIS–HCl (pH 8.1) at room temperature. The gels were stained with 0.1% Coomassie Blue, so that zones of uPA activity occurred as clear lysis zones on a blue background. The bands were quantified densitometrically (Bio IQ Quantifier, B.I. Systems Corporation, Ann Arbor, MI, USA). For controls, parallel samples were loaded on gels without plasminogen. To specifically determine uPA levels also control gels which contained 2 mM amiloride were also used. Amiloride selectively inhibits uPA but does not affect other plasminogen activators (Vassalli, 1987).

### PAI-1–ELISA

Whole cell protein lysates were prepared as described above. PAI-1 protein concentration in cell culture supernatant, Triton extracts (0.01 M HEPES, 0.25 M Sucrose 0.5% v v^−1^, Triton X-100, pH 7.2) of solid DS-sarcomas or DS-sarcoma cells in culture, or in rat serum have been determined by a rat-specific PAI-1 sandwich ELISA (American Diagnostica, Pfungstadt, Germany) according to the manufacturer's recommendations.

### RT–PCR analysis

#### RNA extraction

RNA was isolated from washed cell suspensions or cryosections of solid DS-sarcomas by the guanidinium isothiocyanate method using the Qiagen RNeasy extraction kit (Qiagen, Hilden, Germany) according to the manufacturer's recommendations. RNA content and purity was determined by photometry at 260 *vs* 280 nm.

#### Reverse transcription

1 μg of total RNA was incubated with 50 pmoles of random hexamers for 15 min at 65°C. The mixture was subsequently cooled on ice for 5 min, then the RT-mix was added containing PCR-buffer, 1 U Rnase inhibitor, 15 U MuLV reverse transcriptase, 10 mM MgCl_2_, 1 mM PCR nucleotide mix and double-distilled water up to a volume of 30 μl (all biochemicals from Applied Biosystems, Welterstadt, Germany, except dNTP which was supplied by Roche, Mannheim, Germany). Reverse transcription was carried out in a thermal cycler (Biometra, Göttingen, Germany) for 1 h at 40°C with an additional 5 min-step at 99°C for inactivation of the reverse transcriptase. cDNA was subsequently stored at −20°C.

#### PCR primers

For pseudogene-free amplification of rat β-actin cDNA, previously published primers have been used (Raff *et al*, 1997) (β-actin_sense_: tacaacctccttgcagctcc, β-Actin_antisense_: ggatcttcatgaggtagtctgtc). PAI-1 primers were constructed from published sequences of the rat genome (Genbank accession no. M24067 (Brudzinski *et al*, 1990) PAI-1_sense_: tttgggaaagggttcgcttc PAI-1_antisense_: agtcgttgatgatgaatctgg, intron spanning). Sequence identity of the constructed primers was checked by excising bands from the gel, followed by DNA sequencing. Negative controls with RT (−)-template were carried out to check possible co-amplification of genomic DNA.

#### PCR

Following an initial denaturation period of 3 min at 95°C, PCR was carried out in a thermal cycler (Biometra, Göttingen, Germany) at 95°C/30 s, 60°C 1 min and 72°C/30 s. A final extension period of 72°C/7 min was performed at the end of each PCR reaction. One PCR sample of 25 μl volume contained a mixture of 2.4 mM MgCl_2_ and PCR buffer (Applied Biosystems, Welterstadt, Germany), 0.8 mM dNTP-mix (Roche, Mannheim, Germany) and 0.1 μg cDNA. PCR primers were added up to an amount of 10 pmol each.

#### Fragment detection and evaluation

One per cent agarose gels stained with ethidium bromide were run for 90 min at 60 V. PCR products were visualized on a UV-screen by means of a digital camera and attached hardware (Intas, Göttingen, Germany). Densitometrical evaluation was performed using image processing software (Bio IQ Quantifier, B.I. Systems Corporation, Ann Arbor, MI, USA). To ensure the specificity of the amplification β-actin and PAI-1 band were excised from the gel and sequenced.

#### Semi-quantitative multiplex RT–PCR

For a semi-quantitative analysis of PAI-1 mRNA in solid tumour or isolated DS-sarcoma cells *in vitro*, a non-competitive RT-PCR method in the presence of two pairs of primers for PAI-1 mRNA and for β-Actin mRNA as internal standard was performed. Each PCR-mix was split into six fractions, undergoing PCR for 18, 21, 24, 27, 30 or 33 PCR cycles respectively, followed by agarose gel electrophoresis and densitometry. After plotting the data-points (band intensity) *vs* PCR-cycles, curve fits were performed on the computer in order to generate a PCR-amplification curve for every target. For each amplification graph, a threshold cycle number was extrapolated, corresponding to a threshold value just above the densitometrical background. For every specimen, the calculated difference between the respective threshold cycle of β-actin and PAI-1 was regarded as a measure of PAI-1 gene expression: ΔPAI-1=PAI-1[threshold cycle no.]–β-actin [threshold cycle no.].

#### Statistical analysis

Urokinase plasminogen activator and uPAR expression on the cell surface compared to negative controls were analysed with a paired sample *t*-test. Zymographic uPA activity in the different groups was analysed by a Wilcoxon test for paired samples, and PAI-1 protein concentration in the different groups of samples was compared by the Mann–Whitney test using SPSS 10.0 statistical software.

## RESULTS

### Expression of uPA, uPAR and PAI-1 by DS-sarcoma cells

**Figure 1:**
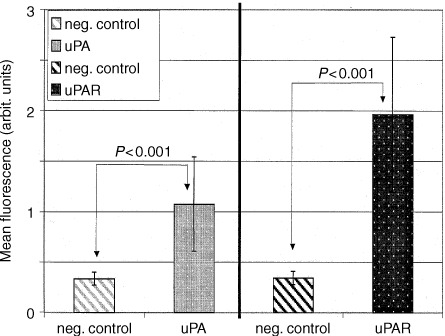
Surface expression of uPA and uPAR on DS-sarcoma cells determined by flow cytometry. There are significant differences in mean fluorescences between uPA or uPAR labelled cells and cells labelled with idiotypic negative controls (*n*=35 per group, means±1 s.d.).

Expression of uPA, PAI-1 and uPAR was examined in solid DS-sarcoma and in DS-sarcoma cells *in vitro*. Surface expression of uPAR and uPA was detected in both DS-sarcoma cells derived from cell culture (Figure 1

) and, to a comparable extent, on cells isolated from solid DS-sarcomas (data not shown). Histological examination of DS-sarcomas demonstrated that the proportion of tumour stroma is quite low in these tumours and the comparison of the uPA and uPAR surface expression by flow cytometry exhibits no significant difference in the mean fluorescence activity between DS-tumour cells isolated from solid tumours and DS-sarcoma cells grown in culture. This indicates that most of uPA and uPAR in the samples derives from tumour cells, rather from leukocytes or stromal cells.

**Figure 2:**
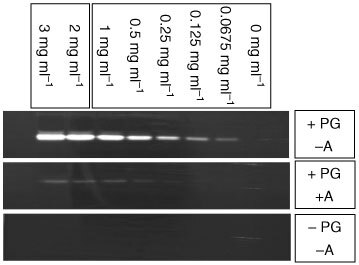
uPA activity in whole cell lysates of DS-sarcoma (0–3 mg ml^−1^ whole cell protein per sample) determined by 1-phase zymography. Lysis zones in the 50 kD region (+PG/−A) are abrogated in gels without plasminogen (−PG/−A) or by inhibition with amiloride (+PG/+A).

The zymography of crude whole cell protein extracts (7 μg/total protein per sample) from solid DS-sarcomas exhibited two plasminogen-dependent proteolytic bands, one with strong, another with weak proteolytic activity in the 50 kDa region. Adding 2 mM/ml amiloride to the SDS–PAGE abrogates the proteolytic activity of the strong lysis zone completely, while the weak band was not influenced at all, indicating that this strong lysis zone represents the proteolytic activity of uPA (Figure 2

). Similar bands were found in protein extracts of DS-sarcoma cells *in vitro* (data not shown).

DS-sarcoma cells also express PAI-1. Plasminogen activator inhibitor type 1 protein could be detected in total protein extracts of solid DS-tumours, but also in whole cell lysate of cultured DS-sarcoma cells (by ELISA). The highest amount of PAI-1 protein was found in cell culture supernatants of DS-sarcoma cells *in vitro*.

Not only PAI-1 protein but also PAI-1 mRNA was found in both RNA extracts from solid DS-tumours and RNA extracts of DS-sarcoma cells *in vitro* by RT–PCR, indicating that PAI-1 is also produced by the tumour cell itself. Since RT–PCR reactions with PAI-1 primers showed no co-amplification of genomic DNA, the possibility of false positive results can thus be disregarded.

### uPA activity in DS-sarcoma cells *in vitro* and in solid tumours

Urokinase plasminogen activator activity was detectable in solid DS-sarcoma and in DS-sarcoma cells *in vitro*. There was no significant difference in band intensity when lysates from solid tumour tissue were compared with whole cell protein lysates from DS-sarcoma cells cultured *in vitro*. This may indicate that in solid DS-sarcomas the tumour cells and not the stromal cells contribute mainly to the proteolytic activity of uPA.

**Figure 3:**
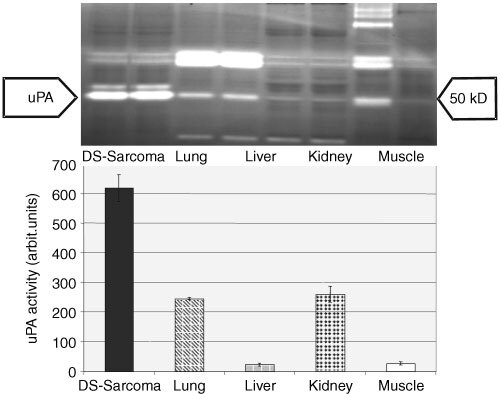
uPA activity in solid DS-sarcomas (assessed by zymography) compared to various rat normal tissues (*n*=6 per group, means±1 s.d.: *P*<0.001, DS-sarcoma *vs* liver and skeletal muscle; *P*<0.01, DS-sarcoma *vs* lung and kidney).

The uPA activity in solid DS-sarcomas was compared to the activity in various normal tissues (lung, skeletal muscle, kidney, liver) by zymography. All normal tissues exhibited some degree of uPA activity. The amount of uPA expression varied significantly between the different tissues with low activity being found in skeletal muscle and liver in contrast to higher activities in kidney and lung. In tumour tissue the uPA activity was much higher than in any type of normal tissue examined. Standardised on 7 μg total protein per sample, the uPA activity in DS-sarcoma was 4–20 fold higher than in the normal tissues examined (Figure 3

).

### The impact of the oxygenation status on uPAR protein expression and overall uPA activity

To examine the impact of the tumour oxygenation status on uPA activity *in vivo*, a set-up for long-term inspiratory hypoxia was used. Immediately after tumour implantation, rats were housed in a hypoxic atmosphere containing 8% O_2_ /92% N_2_. Tumours were compared to those in control animals breathing normal air or 100% O_2_ during the whole period of tumour growth.

In a preceding study the oxygenation of DS-sarcomas under these conditions was determined by computerised pO_2_ histography. Mean median pO_2_ of DS-sarcomas grown in air was about 5 mm Hg and significantly decreased to 2 mm Hg upon inspiration of 92% N_2_/8% O_2_ (Kelleher *et al*, 1997), whereas breathing of pure oxygen led to a significant increase in the mean median pO_2_ up to approximately 80 mm Hg (Thews *et al*, 1997). Various adaptive processes can be expected to take place during long-term exposure of the animals to a hypoxic atmosphere (8% O_2_). Under these conditions, animals showed a polyglobulia with a RBC count of 8.4×10^6^/μl. The increased haematocrit however, results in a higher viscous resistance to flow which might worsen microcirculation under these conditions. Due to adaption, the mean pO_2_ in tumours of chronically hypoxic rats was slightly higher (3 mmHg) compared to acute hypoxia, however still significantly lower compared to air breathing animals. There were no indications for significant improvements of tumour oxygenation by adaptive mechanisms under low oxygen breathing conditions for days.

**Figure 4:**
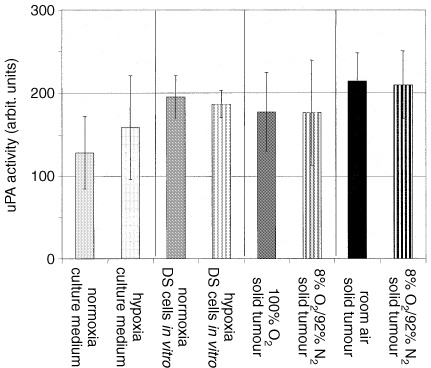
Impact of the oxygenation status on uPA activity in DS-sarcoma cells *in vitro* and in solid tumours: In the supernatant, in whole cell lysates of DS-sarcoma cells and in solid tumours the uPA activity was not significantly different under various conditions of O_2_ availability (culture medium: eight independent samples/group, DS-sarcoma cell lysates: five independent samples/group; solid tumours: inspiratory hypoxia (8% O_2_/92% N_2_) *vs* room air *in vivo*: *n*=50/group, inspiratory hypoxia (8%O_2_/92% N_2_) *vs* hyperoxia (100% O_2_): *n*=14/group; values are means±1s.d.).

Using zymography, the uPA activity in solid DS-sarcomas was compared between solid tumours which were grown under inspiratory hypoxia, normal room air or hyperoxia and in DS-sarcoma cells *in vitro* which were incubated either under hypoxic or normoxic conditions. Under *in vitro* and *in vivo* conditions the uPA activity was comparable (Figure 4

).

The surface expression of uPAR was measured in single cell suspensions derived from solid DS-sarcomas. Mean fluorescence of DS-sarcoma cells was about 5–10 times higher than the background activity of the negative controls and even higher than the signals derived from uPA expression (Figure 1). To evaluate the impact of tumour oxygenation, uPAR expression of tumour cells from solid tumours grown under 8% O_2_/92% N_2_ was compared to uPAR expression in tumours grown in normal room air. There was no enhanced surface expression in the hypoxic group compared to tumours grown in normal air (*n*=50 per group, data not shown). Therefore, a second series of hypoxic tumours (*n*=14; grown under 8% O_2_/92% N_2_ – data not shown) was compared to tumours (*n*=14) grown in rats which were housed in 100% O_2_ to achieve greater differences in the oxygenation. In this latter series, again also no significant difference in uPAR expression could be detected by flow cytometry.

### Impact of tumour volume on uPA activity

**Figure 5:**
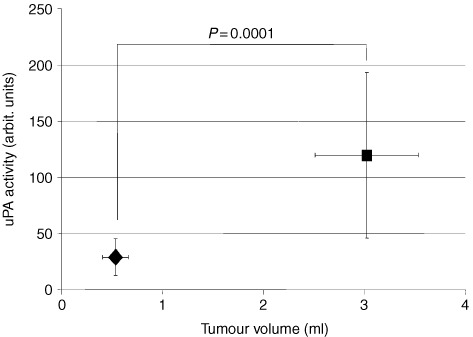
Impact of tumour volume on uPA activity in solid DS-sarcomas. uPA activity measured by one-phase zymography is significantly greater in large tumours (>2 ml) compared to small tumours (<1 ml) (*n*=16 per group, values are means±1 s.d.) (mean pO_2_ in small tumours (mean vol. 0.5 ml): 10.6±3.9 mmHg, hypoxic fraction (pO_2_-values ⩽2.5 mmHg: 33±6%); mean pO_2_ in large tumours (mean vol. 2.5 ml): 2.5±0.4 mmHg, hypoxic fraction (pO_2_-values ⩽2.5 mmHg: 52±3%).

No differences in uPA activity were detected between tumours grown under different O_2_ conditions when the tumour size was equally distributed between the groups. To evaluate the impact of tumour volume, whole cell protein extracts of early stage small tumours (0.5–1 ml tumour volume) were compared to more advanced tumours (volumes of 2.0–3.5 ml). Each group encompassed samples from 16 different tumours. We found a significant, positive correlation between uPA activity and tumour volume. The band intensity standardised on total protein concentration per sample was substantially higher in the larger tumours than in the smaller tumours (*P*=0.0001, Figure 5

). The differences in tumour oxygenation between small and large tumours in this series has not been evaluated, but earlier measurements indicated that the mean pO_2_ is decreased in larger experimental tumours compared to smaller ones.

### Impact of hypoxia on PAI-1 mRNA and protein levels

Plasminogen activator inhibitor type 1, the proteolytic antagonist of uPA, was analysed on the protein and mRNA level. To evaluate the impact of hypoxia on PAI-1 protein expression *in vitro*, PAI-1 protein was measured in supernatant of DS-sarcoma cells *in vitro* grown under hypoxia for 24 h compared to control cultures (normoxic conditions).

**Figure 6:**
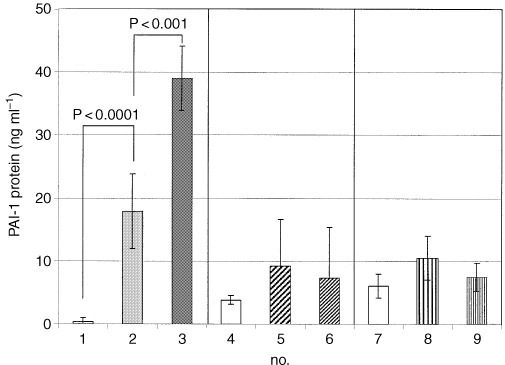
PAI-1 protein concentration in unconditioned culture medium no. 1 compared to culture medium of DS-sarcoma cells grown under normoxia no. 2 or under 24 h exposure to hypoxia no. 3. PAI-1 protein concentration in sera of healthy rats in normal room air no. 4 compared to sera of rats with solid DS-sarcoma housed under inspiratory hyperoxic (100% O_2_) no. 5 or hypoxic (8% O_2_/92% N_2_) no. 6 conditions. PAI-1 protein concentration in solid DS-sarcomas grown under room air no. 7, inspiratory hypoxia (8% O_2_/92% N_2_) no. 8 or hyperoxia (100% O_2_) no. 9 measured by ELISA. (Values are means±1 s.d.).

In supernatants of DS-sarcoma cells after hypoxic challenge a two-fold increase of PAI-1 protein concentration was detected by ELISA in five independent experiments (Figure 6

), 200 000 cells /ml were used. After 24 h, the number of DS-sarcoma cells almost doubled in normoxic controls, while hypoxia delayed cellular growth, so that the number of cells in hypoxic cultures increased only by 10–50% in that time period. The effect of hypoxia may therefore, be even more pronounced when the secretion rate of single cells is considered.

**Figure 7:**
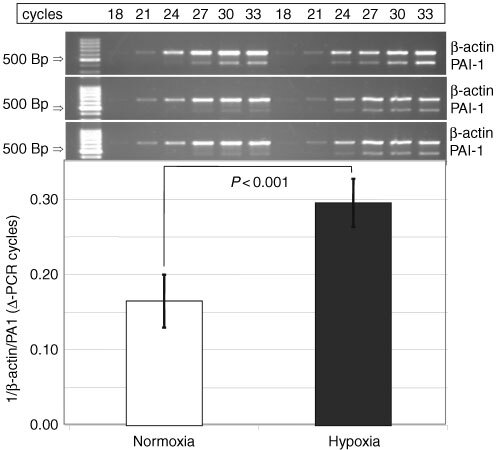
PAI-1 mRNA expression in DS-sarcoma cells *in vitro* after 24 h of hypoxia, compared to control cells under normoxic conditions (calculated as 1/(band int. (β-actin -PAI-1)) (±1 s.d.)). Hypoxia enhances PAI-1 mRNA expresssion (PAI-1 band emerges 2.88 cycles earlier from the background (mean) compared to normoxic controls using β-actin as internal standard).

In the same experiments semi-quantitative multiplex RT–PCR was used to determine whether this effect could additionally be observed on the mRNA level. The amount of PAI-1 mRNA was estimated as a ratio of PAI-1/β-actin mRNA in DS-sarcoma cells *in vitro* immediately after exposure to hypoxia for 24 h. After hypoxic challenge, PAI-1 bands were detected about 2–3 cycles earlier in relation to the internal β-actin standard than in normoxic controls. This effect was reproducible in three independent experiments (Figure 7

).

The impact of the tumour oxygenation status on PAI-1 protein and mRNA concentrations was also analysed *in vivo* using inspiratory hypoxia (8% O_2_/92% N_2_), room air or hyperoxia (100% O_2_) as described above. In each *in vivo*-experiment PAI-1 protein was measured in whole cell protein extracts of solid DS-tumours, and in the serum of rats housed under one of the breathing conditions chosen after tumour inoculation. The individual serum levels of PAI-1 as well as the amount of PAI-1 protein in DS-tumours were quite variable, but there were no significant differences between the median or mean values of both groups. RT–PCR also failed to detect a significant difference in mRNA expression between tumours grown under hypoxic or hyperoxic conditions (data not shown).

## DISCUSSION

We detected uPA, uPAR and PAI-1 in solid DS-sarcomas *in vivo* and in primary cultures of DS-sarcoma cells *in vitro*. In contrast to some other tumour entities (Andreasen *et al*, 1997), in DS-sarcomas the tumour cells themselves generate all three components of the uPA system. Urokinase plasminogen activator-receptor is expressed on the cell surface of DS-sarcoma cells *in vitro* or on single cell suspensions derived from solid tumours, PAI-1 protein and mRNA are detectable in solid tumours and DS-sarcoma cells *in vitro*. Urokinase plasminogen activator activity in whole cell extracts from DS-sarcoma cells *in vitro* and from solid DS-tumours *in vivo* is similar, indicating that the tumour cells are the main source of uPA activity. Therefore, a co-operation between tumour and stromal cells, with different cell types providing different components of the uPA system (as described, for instance, in colon cancer (Sordat *el al*, 1997)), is not necessarily a prerequisite for the functionality of the uPA system in DS-sarcoma. The uPA activity in DS-sarcomas is significantly higher than in all other normal tissues examined, which may be an indication for its functional role in tumour progression.

The question arises as to which mechanism can up-regulate uPA-induced proteolysis under pathological conditions in malignant tumours. The higher expression may be a result ultimately deducible from oncogenic transformation. A wide variety of hormones, growth factors and cytokines are up-regulated as a consequence of oncogenic transformation and are known to regulate the expression of components of the uPA system (Andreasen *et al*, 1997). But a further pathway not directly related to oncogenic alterations can not yet ruled out. The special conditions of the tumour micromilieu, especially tumour hypoxia, are known to have an impact on the expression of a variety of genes and the activity of many gene products (Dachs and Tozer, 2000; Koong *et al*, 2000; Semenza, 2000; Höckel and Vaupel, 2001).

In the present study, incubation of DS-sarcoma cells for 24 h under hypoxia *in vitro* induced a significant up-regulation of PAI-1 protein and mRNA. These results are consistent with the observation that PAI-1 is enhanced in cells lacking a functional von-Hippel-Lindau (VHL) gene (Los *et al*, 1999). pVHL triggers the proteolytic degradation of the hypoxia-inducible factor subunit 1α (HIF-1α) via an ubiquitin-proteasome pathway thereby regulating the transcriptional activity of the most important hypoxia-inducible transcription factor. In pVHL deficient cells, HIF-1 is constitutively upregulated; therefore these cells might behave in some aspects like cells under hypoxic stress.

At a glance, the relation between hypoxia and PAI-1 upregulation seems paradoxical, because the role of PAI-1 as an inhibitor of uPA proteolytic function may also suggest that PAI-1 is predominantly an inhibiting factor of malignant progression in general. However, clinical data contradict this assumption. Far more studies report a positive correlation between high expression of PAI-1 and negative outcome than vice versa (Reuning *et al*, 1998).

Although PAI-1 inhibits proteolytic activity of uPA, this molecule seems to be a marker of aggressiveness in malignant tumours. PAI-1 mRNA is not detectable in low-grade astrocytoma or normal brain tissue by Northern blot, but it is increased in high-grade astrocytomas, and is most pronounced in glioblastoma multiforme (Yamamoto *et al*, 1994). Further biological functions of PAI-1 beyond involvement in proteolysis and extracellular matrix (ECM) degradation may explain this paradoxical finding. There are indications that PAI-1 may be crucial for neoangiogenesis in tumours. Impaired tumour growth and reduced angiogenesis have been described in PAI-1 deficient host mice (Bajou *et al*, 1998).

Plasminogen activator inhibitor type 1 is also related to cell adhesion and motility (Stahl, 1997), and can interfere with the binding between cells and proteins of the extracellular matrix. It reduces the binding between uPAR and vitronectin (VN), and also the binding between integrins of the α_v_ family by masking a somatomedin B-like domain (Chapman, 1997). In neuroblastoma, PAI-1 is positively correlated with metastatic behaviour. Sugiura *et al* (1999) explained this effect by the action of PAI-1 as an anti-detachment factor which interrupts the binding to vitronectin and enhances migration toward fibronectin-rich matrices.

Recent experiments described an anti-apoptotic effect of PAI-1 on tumour and benign cell lines (Kwaan *et al*, 2000). In the present study, DS-sarcoma were seen to be able to survive hypoxic stress, when they had already passed through several cycles of hypoxia and reoxygenation. The relevance of PAI-1 in this phenomenon still has to be determined, but it may be possible that the upregulation of PAI-1 in hypoxia promotes malignant progression and metastasis formation also by additionally increasing resistance to apoptosis (Höckel *et al*, 1999).

*In vitro* assays for invasion showed that not the absolute amount of a single component, but rather the optimal relation between uPA, uPAR and PAI-1 is important for the ability of tumour cells to invade ECM matrixes (for a review see Andreasen *et al*, 1997). Although hypoxia enhances PAI-1 expression *in vitro*, the uPA activity in whole cell lysates or culture supernatants was not diminished.

These *in vitro* effects could not be detected *in vivo* under the conditions described above. The reason for the discrepancy between the *in vitro* and *in vivo* results is not yet clear. Tumour oxygenation *in vivo* is spatially quite heterogeneous, even within a single tumour, and also varies over time. In addition to diffusion-limited (chronic) hypoxia in tumours, changes in vascular patency can generate areas of ischaemic (acute) hypoxia (for a review see Vaupel *et al*, 1989). Therefore, RT–PCR or ELISA are probably not able to detect such differences *in vivo*, because they provide only a ‘global picture’ of protein or mRNA expression at a distinct point in time.

Another reason could be that even globally well-oxygenated tumours exhibit microareas of hypoxia, which can lead to an induction of gene expression. In air breathing animals, solid DS-sarcomas exhibit mean pO_2_ values of about 5 mm Hg, which is apparently low compared to normal tissue, but which is comparable to the oxygenation values of a variety of human tumours measured in clinical situations. Upon breathing 100% O_2_, the mean pO_2_ of DS-sarcomas could be significantly improved, but poorly oxygenated microareas may still exist under such experimental conditions. Kietzmann *et al* (1999) found, that the incubation of cells in an 8% O_2_ atmosphere is able to induce PAI-1 mRNA in rat hepatocytes *in vitro*, which indicates that probably even rather moderate degrees of tumour hypoxia may able to enhance PAI-1 expression. In any case this result also indicates that global parameters (such as PAI-1 or VEGF serum levels) may not be a very reliable marker of tumour hypoxia.

*In vivo*, uPA activity in DS-sarcomas was found to strongly correlate with tumour volume. Small tumours exhibited a significantly lower uPA activity per mg extracted whole cell protein than larger tumours. This may be an indication that uPA activity is not only involved in early events such as basement membrane degradation during transition from *in situ* carcinomas to invasive tumours, but also in later events of malignant progression such as invasion of tissue barriers or intravasation/extravasation of tumour cells.

The increase of uPA activity with tumour size could be a result of an autocrine and/or paracrine positive feedback loop within a solid tumour. TGFβ is a possible candidate for a cytokine involved in such a loop. It is converted from a latent into an active form by plasmin-derived proteolysis, and active TGFβ induces an up-regulation of uPA transcription. However, a hostile tumour micromilieu in larger tumours could also be responsible for this effect. The proportion of hypoxic areas, or areas with poor blood supply, glucose depletion and extracellular acidosis, increases with tumour size in experimental tumours and may also lead to an up-regulation of uPA activity.

In conclusion, these results suggest that the components of the uPA system are up-regulated in rat DS-sarcoma. Micromilieu factors such as hypoxia may contribute to the upregulation of some components of the system, although in this study the *in vitro* results could not be reproduced in the *in vivo* system. The expression pattern of components of the uPA system in different tumour entities is quite variable and the results from one entity could not necessarily be extrapolated to another. But since up-regulation of the uPA system is a relatively common feature in many solid tumours, interferences with this system may be a possible therapeutic approach in at least some entities. It may be possible to combine inhibitors of the uPA system with radiotherapy, as these modalities represent different therapeutic principles and therefore only limited overlapping toxicity should be expected.
